# Metabolic profiling, in vitro propagation, and genetic assessment of the endangered rare plant *Anarrhinum pubescens*

**DOI:** 10.1186/s43141-021-00210-6

**Published:** 2021-07-26

**Authors:** Asmaa Abdelsalam, Ehab Mahran, Kamal Chowdhury, Arezue Boroujerdi

**Affiliations:** 1 Department of Botany, Faculty of Science, Helwan University, Cairo, 11795 Egypt; 2 Department of Chemistry, Claflin University, Orangeburg, SC 29115 USA; 3 Department of Pharmacognosy, Al-Azhar University, Cairo, 11371 Egypt; 4 Department of Biology, Claflin University, Orangeburg, SC 29115 USA

**Keywords:** *Anarrhinum pubescens*, Metabolic profiling, In vitro propagation, Callus culture, Genetic fidelity, NMR spectroscopy

## Abstract

**Background:**

*Anarrhinum pubescens* Fresen. (Plantaginaceae) is a rare plant, endemic to the Saint Catherine area, of South Sinai, Egypt. Earlier studies have reported the isolation of cytotoxic and anti-cholinesterase iridoid glucosides from the aerial parts of the plant. The present study aimed to investigate the chemical profiling of the wild plant shoots as well as establish efficient protocols for in vitro plant regeneration and proliferation with further assessment of the genetic stability of the in vitro regenerated plants.

**Results:**

Twenty-seven metabolites have been identified in wild plant shoots using the Nuclear Magnetic Resonance (NMR) spectroscopy. The metabolites include alkaloids, amino acids, carbohydrates, organic acids, vitamins, and a phenol. In vitro propagation of the plant was carried out through nodal cutting-micropropagation and leaf segment-direct organogenesis. The best results were obtained when nodal cutting explants were cultured on Murashige and Skoog medium with Gamborg B5 vitamins supplemented with 6-benzylaminopurine (BAP) (1.0 mg/L) and naphthaleneacetic acid (NAA) (0.05 mg/L), which gave a shoot formation capacity of 100% and a mean number of shoots of 27.67 ± 1.4/explant. These shoots were successfully rooted and transferred to the greenhouse and the survival rate was 75%. Genetic fidelity evaluation of the micropropagated clones was carried out using random amplified polymorphic DNA (RAPD) and inter simple sequence repeat (ISSR) molecular markers. Jaccard’s similarity coefficient indicated a similarity as high as 98% and 95% from RAPD and ISSR markers, respectively.

**Conclusions:**

This study provides the chemical profiling of the aerial part of *Anarrhinum pubescens*. Moreover, in vitro regeneration through different tissue culture techniques has been established for mass propagation of the plant, and the genetic fidelity of the in vitro regenerated plants was confirmed as well. Our work on the in vitro propagation of *A. pubescens* will be helpful in ex situ conservation and identification of bioactive metabolites.

## Background

*Anarrhinum pubescens* Fresen. (Family: Plantaginaceae) is an endemic perennial plant that grows on granite rocks, in the Saint Catherine region of Egypt [1–4]. The plant is described by the International Union for Conservation of Nature (IUCN) as a rare plant (10.2305/IUCN.UK.2017-3.RLTS.T84119796A84119800.en). The wild plant is distributed in limited narrow areas in only two localities in the Saint Catherine protectorate (Wadi Gebal, Elgabal Elahmar, and Wadi Abu Twita) [5]. The number of plant individuals sharply decreased due to climatic changes and human activities [5–7]. As the wild plant is used in folk medicine for relieving pain. Phytochemical and biological analysis of the plant showed the presence of iridoid compounds which possess cytotoxic and acetylcholinesterase inhibitory activities [8, 9], suggesting the possible application of the plant in developing anticancer and Alzheimer disease relieving drugs. The genus *Anarrhinum* is characterized by the presence of iridoid glycoside compounds. These structurally diverse metabolites are well known for their biological activities, principally, the antimicrobial activities [10, 11]. Iridoid glycosides were isolated from *Anarrhinum orientale* and some of them showed a selective inhibition to hepatitis C virus protease [12, 13].

The application of NMR analysis in metabolic profiling of medicinal plants is a growing field of research. It provides a snapshot for what is included inside the plant tissues and, accordingly, it might help in biomarker discovery in certain plants. In addition, since it gives real information about metabolites included inside the plant cells or tissues, it can be of great importance in distinguishing different plant species sharing the same genus [14].

In vitro propagation strategies for conserving *A. pubescens* have not been studied yet. Traditional methods of propagation via seed germination are unpredictable as it is usually influenced by seasonal changes [15], in the case of *A. pubescens*, only a limited number of plants can be produced due to the rareness of the plant’s seeds. However, biotechnological tools, like tissue culture technology, have proven to provide a reliable method for rapid multiplication of the desired plant species and so conserving its germplasm by establishing tissue banks or increasing intervals between subcultures [16, 17]. Despite the large number of studies on the in vitro propagation of Plantaginaceae plants [18, 19], the application of this in vitro technology to propagate any of the species from genus *Anarrhinum* is still not fully established.

Occasionally, establishment of clonal propagation protocols leads to genetic variations due to stress induced by using plant growth regulators, different media ingredients, or callus intermediate stages [20]. Accordingly, investigation of the genetic stability of the resulting progenies should be considered. Molecular markers techniques such as RAPD, ISSR, and restriction fragment length polymorphism (RFLP) have been increasingly utilized to evaluate the genetic fidelity of the propagated plants, and their common use for this purpose has been well documented [21, 22].

Plants serve as a substantial source of bioactive metabolites. Plants belong to Plantaginaceae family are sources for many biologically active compounds, principally, the antimicrobial compounds [23, 24].

Here, we report the metabolic profiling, in vitro propagation, and callus induction for genus *Anarrhinum*, which could be an important basic reference for any further research concerned with chemical analysis and mass propagation of the *A. pubescens* plant or other species sharing the same genus. Our study displays efficient protocols for in vitro regeneration and callus induction of *A. pubescens* as reliable methods for medium to long-term conservation of this endangered rare plant.

## Methods

### Plant material

Seeds and aerial parts (leaves and stems) of *Anarrhinum pubescens* were collected from St. Catherine, Egypt in April 2015. The plant was authenticated by the staff members of the St. Catherine protectorate. Plant aerial parts were used for the metabolic profiling of the plant, whereas seeds were used for the tissue culture experiments.

### Chemicals

Tissue culture media (Murashige and Skoog medium with Gamborg B5 vitamins) and growth regulators (6-benzylaminopurine, kinetin, naphthaleneacetic acid and 2, 4 dichlorophenoxy acetic acid) were purchased from Phyto-Technology Laboratories (Shawnee Mission, KS, USA). Ethanol, methanol, chloroform, and TMSP buffer components were purchased from Sigma-Aldrich (St. Louis, MO, USA).

### Metabolites extraction from the wild plant

Six individuals from the wild plant were selected randomly, air dried, ground, and homogenized under liquid nitrogen. From each sample, 20 mg dry weight was used for the extraction processes. Metabolites were extracted using 2:2:1.8 methanol: chloroform: water (V/V/V) [25] and resulted in separated polar and nonpolar liquid phases upon centrifugation for 10 min at 2000×*g* and 4 °C. The polar extract (upper phase) was dried using a Centrivap vacuum concentrator for 24 h at 30 °C.

### NMR sample preparation and data collection

The dried polar extract was rehydrated in 620 μL NMR buffer (100 mM sodium phosphate buffer (pH 7.3), 1 mM TMSP (internal standard,3-(trimethylsilyl) propionic-2,2,3,3-d4 acid, CAS: 24493-21-8), and 0.1% sodium azide, in 99.9 atom % D_2_O).

All data were collected using a spectral width of 16.0 ppm and 64K points resulting in an acquisition time of 2.9 s. The first increment of the presat-noesy spectra was collected with 120 scans, 4 dummy scans, 3 s relaxation delay, and on-resonance pre-saturation at the residual water frequency for solvent suppression. The 90 ° pulse widths were measured for each sample using the automatic pulse calculation experiment (pulsecal) in TopSpin 2.1.1 (BrukerBioSpin, Billerica, MA). Two dimensional ^1^H-^13^C HSQC data were collected at 700 MHz using a Bruker hsqcedetgpsisp2.2 pulse sequence. The ^1^H was observed in the F2 channel with a spectral width of 11 ppm while the ^13^C was observed in the F1 channel with a spectral width of 180 ppm.

### Metabolite identification and quantification

Polar metabolites from the aerial part of the plant were identified by comparing the ^1^H data with the 700 MHz database of metabolite standards in Chenomx NMR Suite (Chenomx Inc., Edmonton, Alberta, Canada). All identified metabolites were authenticated by comparing the obtained 1D and 2D spectra with the data represented in Human Metabolome Database (HMDB).

### In vitro propagation protocol

#### Culture conditions and seedling development

*Anarrhinum pubescens* seeds were rinsed three times with distilled H_2_O for 5 min each, then surface sterilized by dipping in 95% ethanol for 1 min, followed by disinfection with 20% commercial Clorox® solution containing one drop of Tween 20® for 20 min and finally washed four times with sterile distilled H_2_O for 5 min each under aseptic conditions. Seeds were germinated by culturing on full strength Murashige and Skoog medium with Gamborg B5 vitamins (MS-B5) [26, 27] in magenta vessels using six seeds per vessel. The culturing media contained 30 g/L sucrose, adjusted to pH ~ 5.7 and solidified with 2 g/L phytagel (St. Louis, MO, USA) before autoclaving at 121 °C for 20 min. Cultures were incubated at 25 °C under 16 h photoperiod provided by cool white fluorescent lamps for 4 weeks.

### Shoot multiplication

Seedlings (4-week-old) were defoliated and cut into 0.8–1.0 cm nodal segments (0.5 cm down from the nodes) prior to being vertically inoculated in culture medium with an immersion length from the bottom 0.3 cm of the bottom internode (nodes were kept above the surface of the medium). Nodes were cultured on full-strength MS-B5 medium supplemented with 30 g/L sucrose and containing different concentrations of 6-benzylaminopurine (BAP) (0.5, 1.0, 2.0, 3.0, and 4.0 mg/L) or kinetin (Kn) (0.5, 1.0, 2.0, 3.0, and 4.0 mg/L) plus a fixed concentration of naphthaleneacetic acid (NAA) (0.05 mg/L). The medium was solidified using 2.0 g/L phytagel. Nodal cutting explants were placed in magenta vessels using three explants per vessel. At least nine replicates were used per treatment and all experiments were repeated three times. All cultures were incubated for a 16-h photoperiod at 25 °C in a completely randomization design. Six weeks later, the cultures were screened for percentage of shoot formation (%) =$\frac{\mathrm{No}.\kern0.5em \mathrm{of}\ \mathrm{explants}\ \mathrm{that}\ \mathrm{formed}\ \mathrm{shoots}\ }{\mathrm{Total}\ \mathrm{No}.\mathrm{of}\ \mathrm{explants}} \times 100$ and mean number of shoots = $\frac{\mathrm{Total}\ \mathrm{No}.\kern0.5em \mathrm{of}\ \mathrm{shoots}\ }{\mathrm{Total}\ \mathrm{No}.\kern0.5em \mathrm{of}\ \mathrm{explants}\ }$and the results were statistically analyzed.

### Rooting and acclimatization

Developed shoots (3−4 cm) derived from nodal cuttings cultured on full strength MS-B5 medium supplemented with BAP (1.0 mg/L) and NAA (0.05 mg/L) were separated and transplanted into full strength hormone free MS-B5 basal medium with and without 0.1 mg/L IAA. Twenty shoots were transferred to each rooting media. After 30 days of incubation, cultures were examined for the percentage of root formation (%) =$\frac{\mathrm{No}.\kern0.5em \mathrm{of}\ \mathrm{explants}\ \mathrm{formed}\ \mathrm{roots}\ }{\mathrm{Total}\ \mathrm{No}.\kern0.5em \mathrm{of}\ \mathrm{explants}} \times 100.$ Well rooted shoots were removed from the culture vessels, washed thoroughly with tap water, and transferred to 4-cellplastic trays containing the sterilized mixture of Lowes Miracle-Gro All Purpose Potting mix and Vermiculite at 3:1 volumetric ratio (Lowe’s Company, USA) and completely enclosed in transparent plastic bags. Afterwards, plantlets were transferred to the greenhouse and maintained at 27 ± 2 °C under programmed watering.

### Statistical analysis

For in vitro propagation, a completely randomized experimental design was performed. All experiments were repeated three times, each time using at least 9 replicates (for in vitro propagation) or 12 replicates (for callus induction) per treatment. Each replicate was represented by 1 plate/4 explants. All replicates came from different seeds. Collected data were subjected to analysis of variance (ANOVA) and means were compared through Fisher pairwise comparison (*p* ≤ 0.05). Statistical analyses were accomplished using Minitab® software.

### Genetic fidelity assessment

Genomic DNA of mother plants and eight replicates from the in vitro propagated plantlets was isolated using E.Z.N.A. DNA isolation kit as per manufacturer’s instructions (Promega, Madison, WI, USA). DNA quality and quantity were determined by recording the ratio of absorbance at 260 to 280 nm using a NanoDrop-1000® Spectrophotometer (NanoDrop Technologies, Wilmington, DE, USA). DNA samples were diluted to 50 ng/μl. A total of 17 RAPD and 25 ISSR primers (Eurofins MWG Operon LLC, Louisville, KY, USA) were initially screened to evaluate the genetic stability of the micropropagated plants; then only 10 RAPD and 10 ISSR primers were chosen based on their reproducibility to generate clear, scorable bands. Amplification reaction was carried out in a 25-μl mixture containing 50 ng of template DNA, 5 μl 5X*Taq* buffer (Promega, Madison, WI, USA), 2.5 μl dNTPs, 1 U *Taq* polymerase (Promega, Madison, WI, USA), 25 mM MgCl_2_, and 1 μl primer in a thermal cycler (Eppendorf Mastercycler®, Hamburg, Germany). PCR conditions were programmed as follows: initial denaturation at 94 °C for 4 min followed by 40 cycles of 1 min denaturation at 94 °C, 30 s annealing at the specified temperature for each primer, and 1 min extension at 72 °C and then final extension for 10 min at 72 °C. Amplification products were resolved by gel electrophoresis on 1.5% agarose gel running for 1 h at 105 V in 1X TAE; DNA bands were stained with ethidium bromide. Resulting bands were detected and photographed by Gel Doc XR+ imager® (Bio-Rad, Hercules, CA, USA). Bands sizes were determined by the aid of 1 kb plus DNA ladder (Fisher Scientific, USA). Quantity One Software (BioRad Laboratories, Hercules, CA, USA) was used to analyze the gel profiles. Data matrix was constructed based on scoring clear, reproducible bands into a binary data sheet (1 for presence and 0 for absence). Jaccard’s similarity coefficient [28] was calculated using community analysis package 1.14 (CAP) by Henderson and Seaby [29].

## Results

### Metabolic profiling of the wild plant shoots

**Fig. 1:**
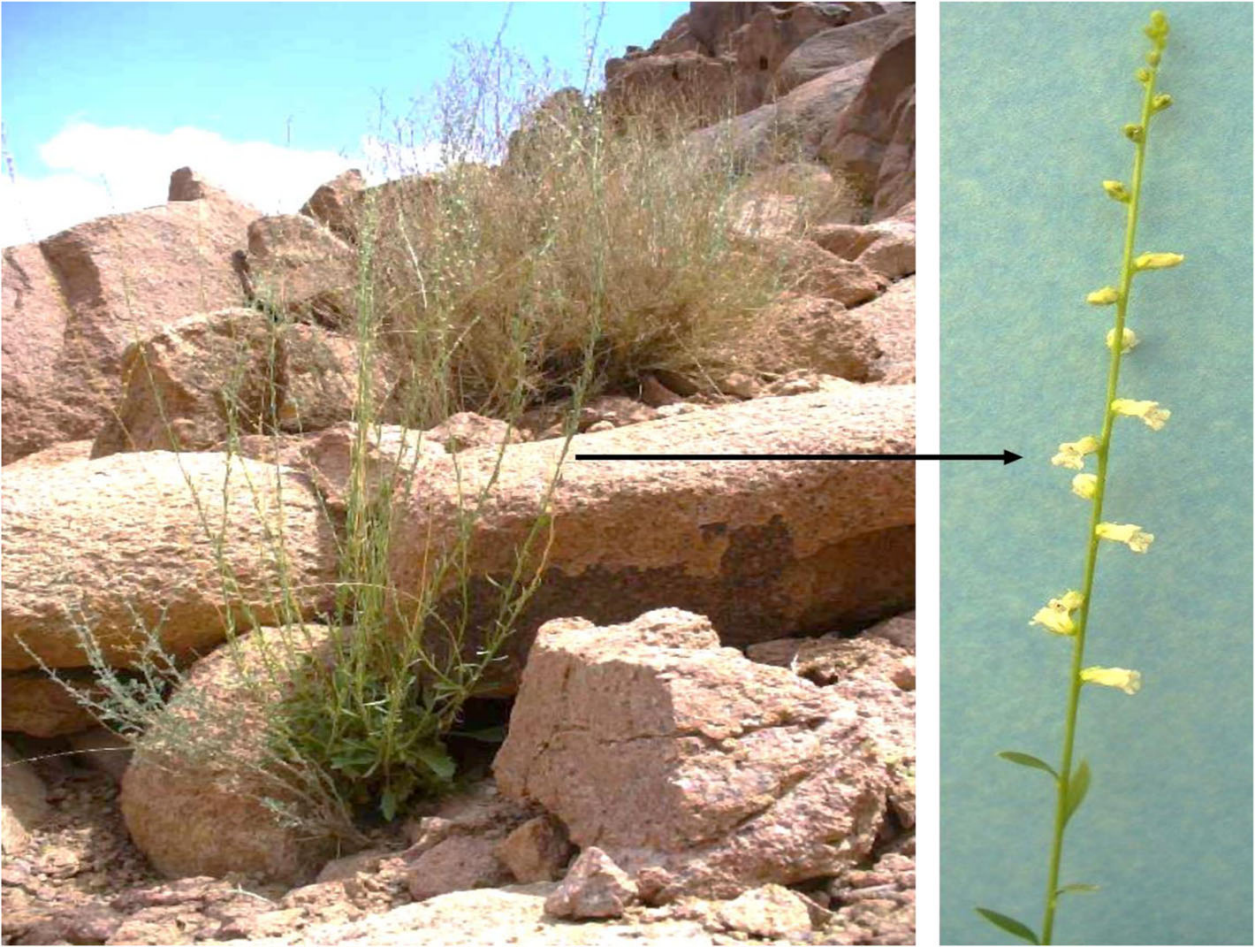
*Anarrhinum pubescens* wild plant

**Fig. 2:**
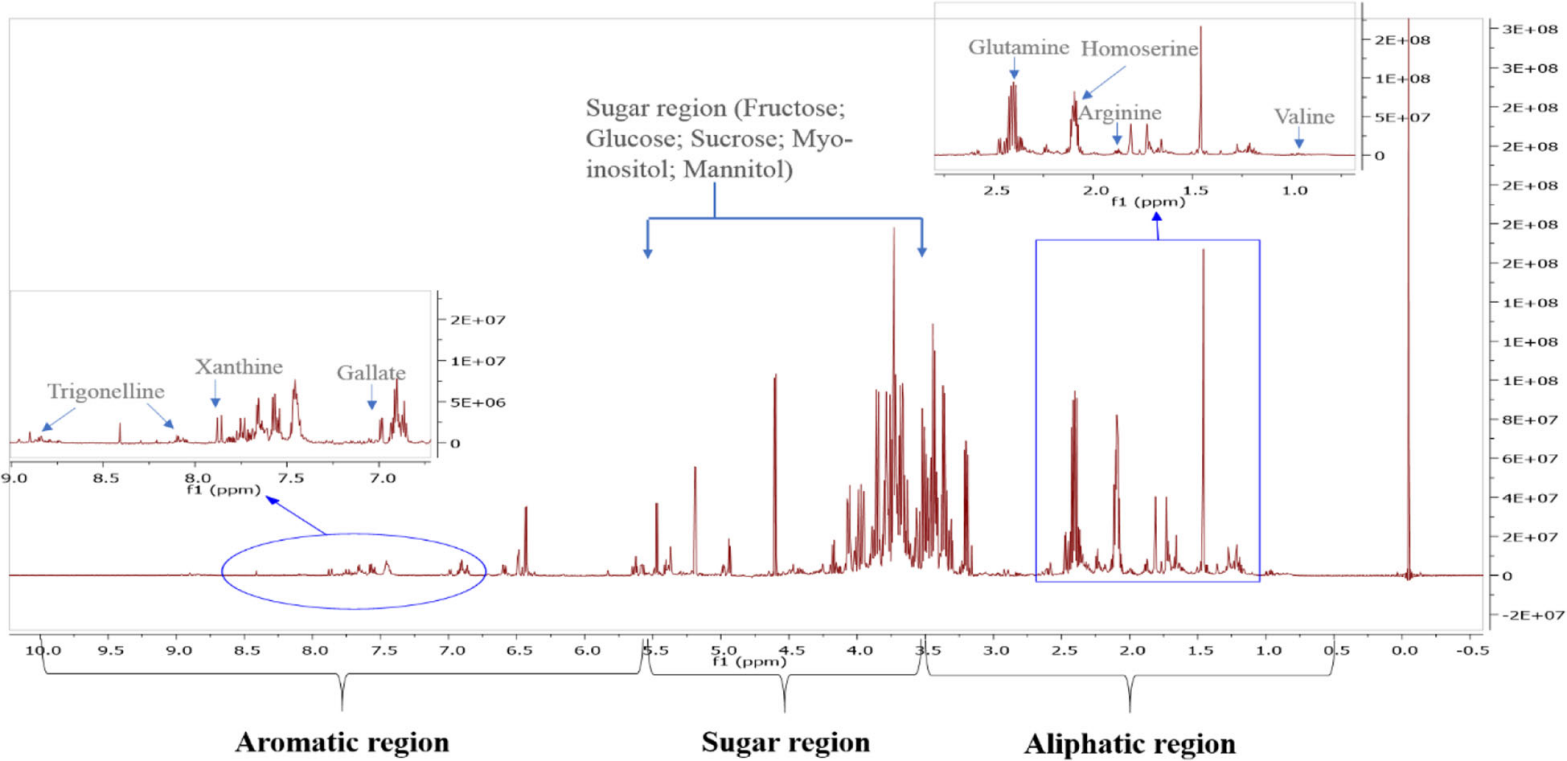
^1^H spectra of the polar extract of *Anarrhinum pubescens*

The peaks from the ^1^H spectra were assigned using Chenomx software (8.6) and its 700 MHz database. For verification, ^1^H-^13^C HSQC data were analyzed and compared with^1^H-^13^C HSQC data available online in Human Metabolome Database (HMDB). Twenty-seven metabolites (Table 1) were successfully identified in the polar extract of the wild plant shoots (Fig. 1). The identified compounds include organic acids, vitamins, amines, amino acids, sugars, sugar alcohols, alkaloids, and a phenol. The aliphatic region between δ 0.5 and 3.5 ppm was characterized by the presence of essential and non-essential amino acids like isoleucine, valine, arginine, and betaine. In the sugar region (Fig. 2), δ 3.5–5.5 ppm, we identified the presence of myo-inositol, sucrose, mannitol, fructose, and glucose. Xanthine, gallate, and trigonelline were identified in the aromatic region δ 5.5.5–10.0 ppm. Also, vitamins and organic acids like choline and acetic acid were identified.

**Table 1:** list of metabolites identified in the polar extract of *A. pubescens* shoot

–	Compound name	^13^C (ppm) from HSQC	^1^H (ppm) (functional group or specific H, multiplicity)	Coupling constant *J* (Hz)
1	Acetate	26.14 (CH_3_)	1.90(CH_3_, s)	–
**2**	Alanine	18.93 (C^β^)	1.46 (H^β^, d)	7.25
		53.5 (C^α^)	3.77(H^α^, q)	7.23
**3**	Arginine	26.6(C^γ^)	1.64 (H^γ^, m)	–
		26.5(C^β^)	1.88 (H^β^, m)	–
		57.03(C^α^)	3.75 (H^α^, m)	–
**4**	Asparagine	37.5 (C^β^)	2.94 (H^β^, dd)	4.16,16.8
			2.85 (H^β^, dd)	7.74,16.8
**5**	Betaine	56.23 (CH_3_)	3.24 (CH_3_, s)	–
		69.19 (CH_2_)	3.89 (CH_2_, s)	﻿–
**6**	Choline	56.81 (C^γ^)	3.19 (H^γ^, s)	–
		70.38 (C^β^)	3.50 (H^β^, m)	–
**7**	Dimethylamine	37.0 (CH_3_)	2.71(CH_3_, s)	–
**8**	Formate	–	8.44 (CH, s)	–
**9**	Fructose	71.95	3.98 (^5^CH, m)	–
		78.20	4.12 (^3^CH, m)	–
**10**	Fumarate	138.12 (CH)	6.51 (CH, s)	–
**11**	Gallic acid	7.02 (CH)	112.119 (CH, s)	–
**12**	Glucose	74.41 (^2α^CH)	3.52 (^2α^CH, m)	–
		72.58 (^4^CH)	3.40 (^4^CH, m)	–
		63.70 (^6^CH)	3.74 (^5β^CH, m	–
		98.92 (^1α^CH)	4.66 (^1α^CH, d)	7.86
**13**	Glutamine	29.08 (C^β^)	2.14 (H^β^, m)	–
		57.09 (C^α^)	3.76 (H^α^, t)	6.40
**14**	Glycine	44.34 (C^α^)	3.55 (H^α^, s)	–
**15**	Homoserine	35.12 (C ^β^)	2.02 (H^β^, m)	–
16	Isoleucine	17.54 (C^γ^)	1.00 (H^γ^, d)	7.01
		26.83 (C^γ^)	1.24 (H^γ^, m)	–
17	myo-Inositol	77.0	3.3 (CH,t)	
		73.8	3.6(CH, t)	
18	Mannitol	65.98	3.85 (CH_2_, dd)	
		73.48	3.76(CH, d)	
19	Pyroglutamate	27.9 (C^β^)	2.4 (H^γ^,m)	–
		60.9 (C^α^)	4.2(H^α^, dd)	9.0, 5.9
20	Pyruvate	29.33 (CH_3_)	2.36 (CH_3_, s)	–
21	Succinate	37.14 (CH_2_)	2.39 (CH_2_, s)	–
22	Sucrose	72.1 (^3^CH)	3.48 (^3^CH, m)	–
		64.2 (^1^CH_2_)	3.69 (^1^CH_2_, s)	–
		79.8 (^3^CH)	4.22 (^3^CH, d)	8.82
		95.1 (^1^CH)	5.42 (^1^CH, d)	3.90
**23**	trans-Aconitate	40.4 (CH_2_)	3.43 (CH_2_, s)	–
		134.0 (CH)	6.56 (CH, s)	–
**24**	Trigonelline	148.2 (^2^CH)	9.10 (CH, s)	–
		147.4 (^4,6^CH)	8.84 (CH, t)	
		130.5 (^5^CH)	8.08 (CH, t)	8.80 7.37
		51.05 (^1^CH_3_)	4.4 (CH_3_, s)	
**25**	Trimethylamine	47.3 (CH_3_)	2.87 (CH_3_, s)	–
**26**	Valine	20.83 (C^γ^)	0.97 (H^γ^, d)	7.02
		19.53 (C^γ^)	1.03 (H^γ^, d)	7.00
**27**	Xanthine	140.3 (CH)	7.90 (CH, s)	–

### Micropropagation via nodal cutting

**Fig. 3:**
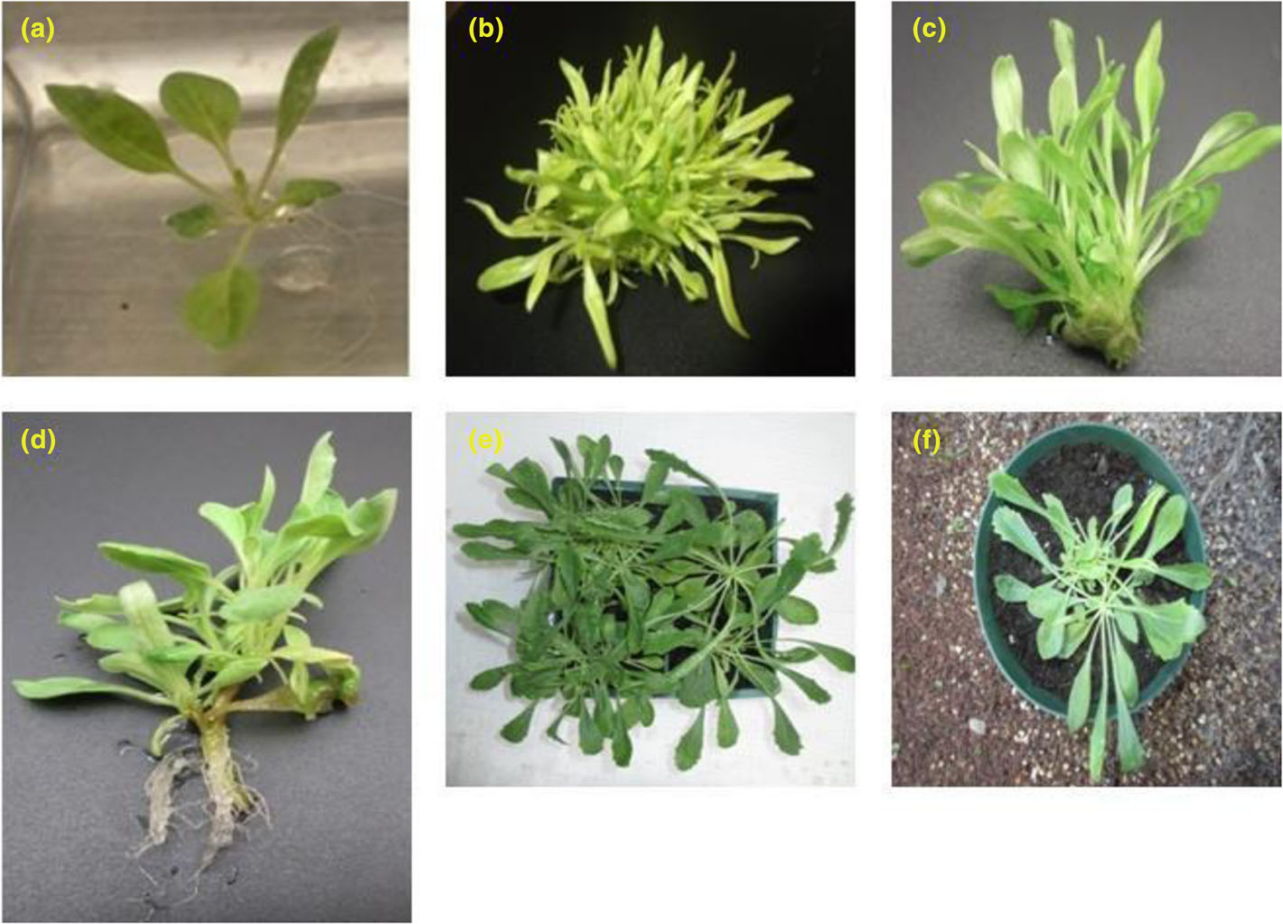
Developmental stages of the in vitro propagation of *Anarrhinum pubescens*. A 4-week-old seedling (**a**), micropropagated shoots using BAP 1 mg/L + NAA 0.05 mg/L (**b**), organogenic shoots using BAP 0.05 mg/L + NAA 0.05 mg/L (**c**), rooted-shoots using MS-B_5_ with 0.1 IAA mg/L (**d**), 10-week-old greenhouse-acclimatized plantlets (**e, f**)

Nodal cuttings were excised from seedlings (Fig. 3a) and cultured on MS-B_5_ medium supplemented with BAP or Kn, at different concentrations (0.5, 1.0, 2.0, 3.0, and 4.0 mg/L). Statistical analysis of data collected after 6 weeks of incubation revealed that, BAP concentrations were superior in adventitious shoots induction and proliferation (Fig. 3). Different BAP concentrations showed 100% capacity rate (all explants produced shoots). On the other hand, Kn treatments showed an induction rate range of 71.1–100%. A higher mean number of shoots (27.67 ± 1.4, 23.19 ± 0.5 shoots/explant) were recorded with 1.0 and 2.0 mg/L BAP, respectively (Table 2). Mean number of shoots decreased from two to five times by using Kn instead of BAP.

**Table 2:** Effect of different cytokinin (BAP and Kn) concentrations in combination with 0.05 mg/L NAA on shoot multiplication from different explant sources of *A. pubescens*

Growth regulators (mg /L)			Explant source			
BAP	Kn	NAA	Nodal cuttings		Leaf segments	
			Shoot response^*^ (%)	No. of shoots^*^	Shoot response* (%)	No. of shoots^*^
0.5	–	0.05	100^a^	13.85 ± 0.7^c^	81.5^ab^	9.3 ± 0.6^a^
1	–	0.05	100 ^a^	27.67 ± 1.4^a^	88.8^ab^	7.1 ± 0.7^b^
2	–	0.05	100 ^a^	23.19 ± 0.5^b^	77.8^b^	7.4 ± 0.2^b^
3	–	0.05	100 ^a^	13.33 ± 1.6^c^	48.2^c^	3.7 ± 0.6^c^
4	–	0.05	100 ^a^	12.67 ± 1.7^c^	85.2^ab^	8.2 ± 0.2^ab^
–	0.5	0.05	96.3 ^a^	3.6 ± 0.2^e^	74^c^	1.2 ± 0.6^d^
–	1	0.05	100 ^a^	5.8 ± 0.6^de^	7.4^d^	0.4 ± 0.3^d^
–	2	0.05	100 ^a^	8.3 ± 1.7^d^	66.7^c^	2.3 ± 0.2^cd^
–	3	0.05	74.1^b^	5.8 ± 2.4^de^	70.4^c^	1.2 ± 0.4^d^
–	4	0.05	92.6^a^	5.1 ± 0.8^de^	90.3^a^	6.5 ± 1.4^b^

*Values represent mean ± SEM; numbers in columns followed by same letters are not significantly different according to Fisher pairwise comparison (*p* ≤ 0.05)

### Micropropagation via leaf explant

In vitro regeneration using leaf explants was performed by culturing the seedling-derived leaf segments (Fig. 3a) on the same plant growth regulator types and concentrations as used in nodal cutting culture. BAP was significantly superior to Kn in adventitious shoots induction and proliferation. The maximum percentage rate (88.8–81.5 %) of adventitious shoots induction was produced at a wide range of BAP (0.5, 1.0, and 4.0 mg/L), but only at one concentration from Kn (4.0 mg/L). The highest mean number of shoots (9.30 ± 1.4 shoots/explant) was obtained by using 0.5 mg/L BAP (Fig. 3b, c, Table 2). A lower mean number of shoots was recorded with 1.0 mg/L Kn. Within Kn concentrations, 4.0 mg/L was superior in shoots induction (90.3%) and multiplication (6.5 ± 1.4 shoots/explant).

### Rooting and acclimatization

**Fig. 4:**
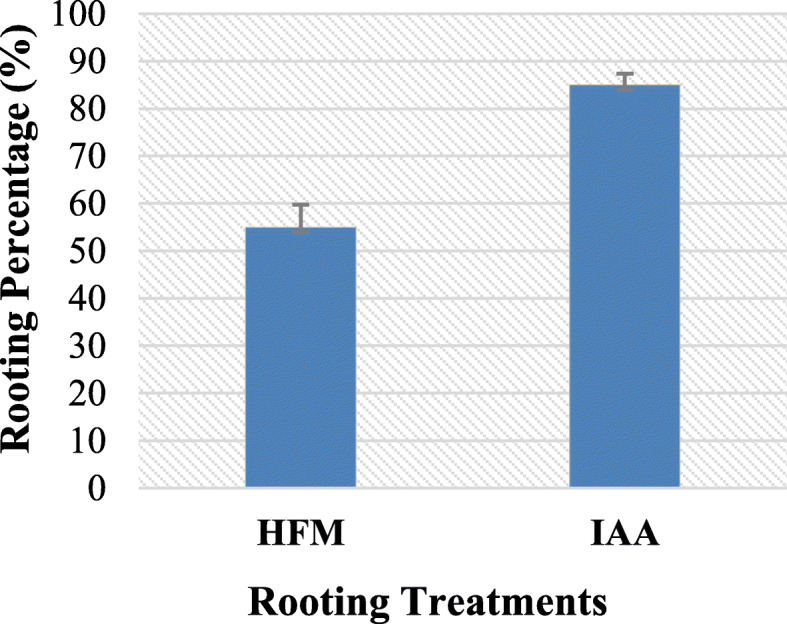
Adventitious rooting percentage on MS-B_5_ hormone free medium = HFM; MS-B_5_ with 0.1 IAA mg/L = IAA

Healthy regenerated shoots derived from nodal cuttings and induced on MS-B_5_ medium with 1 mg/L BAP were examined for rooting capacity. Rooting capacity test was carried out on MS-B_5_ hormone-free medium or MS-B_5_ medium supplemented with 0.1 IAA mg/L. Rooting response (a percentage of root formation) was higher (85%) in IAA-treated samples (Fig. 3d and Fig. 4). IAA-treated plantlets were transferred to the greenhouse and showed 75% survival after 10 weeks of acclimatization (Fig. 3e, f).

### Genetic fidelity assessment

As a rule of thumb, maintaining the genetic uniformity of the propagated plants is an essential prerequisite for most of the mass propagation experimentation. So, in this study, RAPD and ISSR molecular markers were used to evaluate the genetic fidelity of the micropropagated clones. Genomic DNA was isolated from both mother plants and those obtained from the best propagation medium (MS-B_5_ media with 1 mg/L + 0.05 mg/L NAA) and analyzed by PCR-based molecular markers to trace the occurrence of any polymorphism. Ten out of 17 RAPD and 10 out of 25 ISSR primers were selected to accomplish the study.

**Fig. 5:**
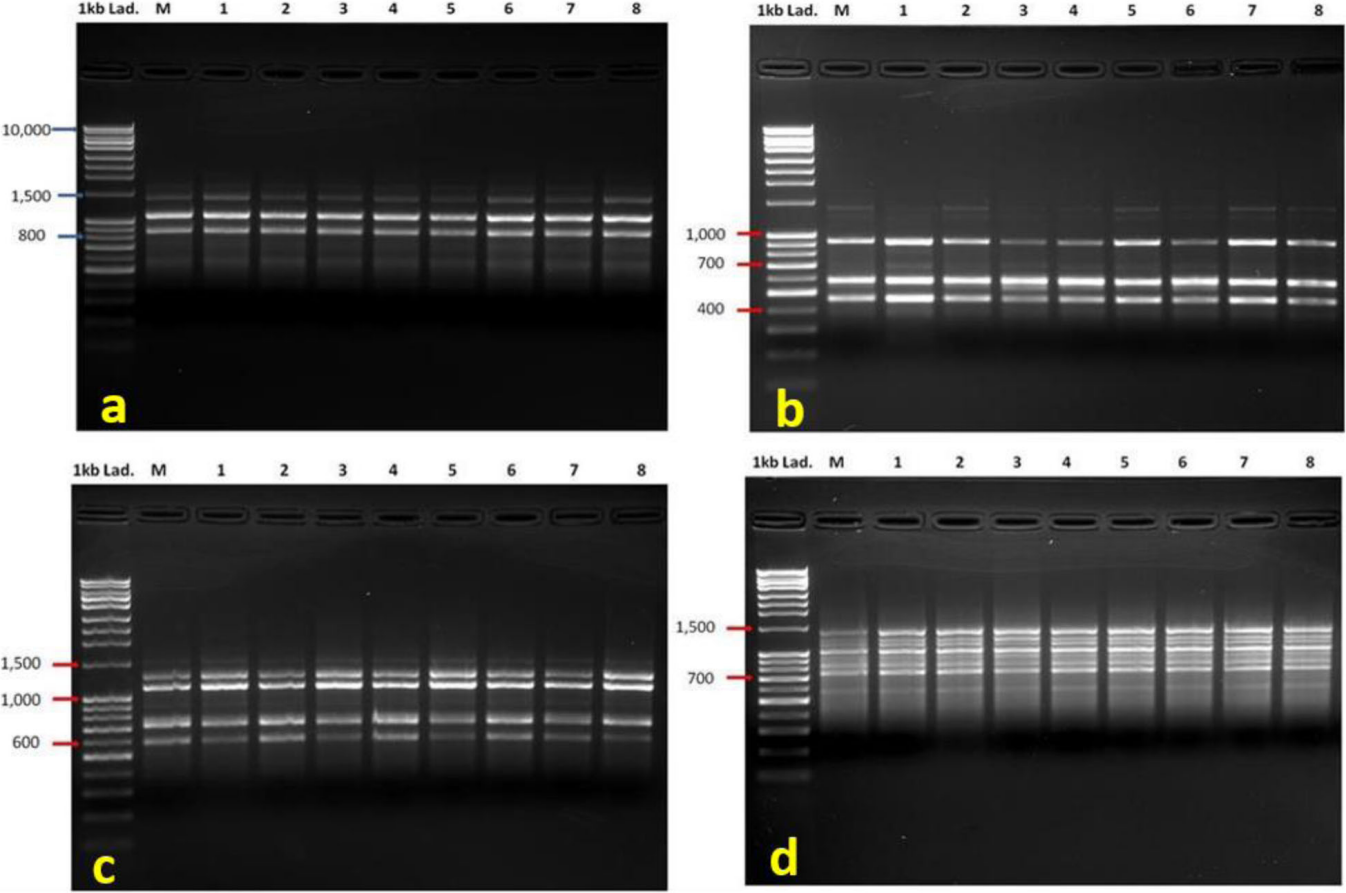
DNA amplification profile of mother and in vitro propagated plants of *A. pubescens* generated by RAPD and ISSR primers. RAPD primer OPA-03 (**a**), RAPD primer OPI-07 (**b**), ISSR primer UBC-860A (**c**), and ISSR primer R(CA)7 (**d**). 1st lane is 1 kb DNA ladder, lane M-mother plant, lane 1–8 in vitro propagated plants

The 10 RAPD primers generated a total of 63 clear, distinct, and reproducible bands with an average of 6.3bands/primer and range size 3860–408 PB (Table 3). Eight out of the chosen 10 RAPD primers showed 100% monomorphism. The highest percentage of polymorphism was 25% recorded by the primer OPI-07 (Fig. 5b). The highest number of bands was generated by primer OPA-04 which gave a total of 13 bands. Pair-wise analysis indicated a similarity index ranged from 0.97 to 0.98 between the mother and the in vitro raised plants (Table 4).

**Table 3:** List of RAPD and ISSR primers’ names, sequences, annealing temperatures, bands sizes range, and number of bands amplified including monomorphic (MB) and polymorphic bands (PB)

Molecular marker	Primer name	Primer sequence 5′ to 3′	Temp. (^°^C)	Size range (bp)	Total amplified bands	MB	PB
RAPD	OPA-03	AGTCAGCCAC	39.5	1787–921	4	4	0
	OPA-04	AATCGGGCTG	39.5	2667–408	13	12	1
	OPH-07	CTGCATCGTG	39.5	2200–613	6	6	0
	OPI-07	CAGCGACAAG	39.5	1926–617	4	3	1
	OPK-07	AGCGAGCAAG	39.5	1788–690	11	11	0
	OPM-07	CCGTGACTCA	39.5	3860–840	7	7	0
	OPO-07	CAGCACTGAC	39.5	1642–538	4	4	0
	OPS-07	TCCGATGCTG	39.5	1813–516	4	4	0
	OPW-07	CTGGACGTCA	39.5	2114–625	6	6	0
	OPY-07	AGAGCCGTCA	39.5	1919–621	4	4	0
	Total				63	61	2
	Range			3860–408	13–4	12–4	2–0
	Average				6.3	6.1	0.2
ISSR	UBC-842C	GAGAGAGAGAGAGAGACG	59.9	2013–510	7	7	0
	UBC-842T	GAGAGAGAGAGAGAGATG	57.6	1617–567	9	7	2
	UBC-846A	CACACACACACACACACAAT	55.3	1717–520	4	3	1
	UBC-849C	GTGTGTGTGTGTGTGTCA	57.6	2682–678	13	12	1
	R(CA)7	GCACACACACACACA	53.4	1410–580	8	8	0
	UBC-834C	AGAGAGAGAGAGAGAGCT	57.6	4220–734	5	4	1
	UBC-834T	AGAGAGAGAGAGAGAGTT	55.3	1111–464	3	3	0
	UBC-848A	CACACACACACACACACAAG	57.6	1450–687	3	3	0
	UBC-860A	TGTGTGTGTGTGTGTGAA	55.3	2170–730	5	4	1
	(AC)8TG	ACACACACACACACACTC	57.6	2738–1540	9	8	1
	Total				66	59	7
	Range			4220–464	13–3	12–3	1–0
	Average				6.6	5.9	0.7

**Table 4:** Jaccard’s similarity coefficient for mother and in vitro regenerated plants of *Anarrhinum pubescens* based on RAPD markers

	M	I1	I2	I3	I4	I5	I6	I7	I8
M	1								
I1	0.98	1							
I2	0.97	0.98	1						
I3	0.97	0.98	1	1					
I4	0.98	1	0.98	0.98	1				
I5	0.98	1	0.98	0.98	1	1			
I6	0.97	0.98	1	1	0.98	0.98	1		
I7	0.98	1	0.98	0.98	1	1	0.98	1	
I8	0.98	1	0.98	0.98	1	1	0.98	1	1

The ISSR-based genomic analysis gave a total of 66 bands where 59 of them were monomorphic (Table 3). Band sizes ranged from 4220 to 464 PB. The maximum number of amplified fragments (13) was recorded by UBC-849C primer, while primers UBC-834T and UBC-848A produced only 3 bands each. Jaccard’s similarity coefficient analysis indicated a similarity index of 0.92–0.95 between the mother and the in vitro regenerated plants (Table 5).

**Table 5:** Jaccard’s similarity coefficient for wild and in vitro regenerated plants of *A. pubescens* based on ISSR markers

	M	I1	I2	I3	I4	I5	I6	I7	I8
M	1								
I1	0.95	1							
I2	0.92	0.94	1						
I3	0.94	0.98	0.95	1					
I4	0.94	0.95	0.98	0.94	1				
I5	0.94	0.98	0.92	0.97	0.94	1			
I6	0.92	0.94	0.97	0.92	0.98	0.95	1		
I7	0.94	0.98	0.92	0.97	0.94	1	0.95	1	
I8	0.94	0.95	0.98	0.94	1	0.94	0.98	0.94	1

## Discussion

### Metabolic profiling of the wild plant shoots

In this study, we successfully identified 27 metabolites in the polar extract of the wild plant shoots which possess nutritional and medicinal values. Here, amino acids like isoleucine, valine, alanine, arginine, glutamine, and betaine have been identified. These amino acids have been previously reported in other members of the family Plantaginaceae, e.g., alanine, arginine, glutamine, isoleucine, and valine have been identified in *Plantago ovata* leaves [30]. Also, arginine, glutamine, isoleucine, and glycine were the most abundant amino acids in *Veronica teucrium* [31]. Isoleucine and valine are essential branched amino acids, which cannot be de novo synthesized by the human body and should be supplied in food. Isoleucine and valine have been reported to stimulate muscle protein synthesis. Isoleucine has been proved to encourage the uptake of glucose in muscle and glucose oxidation in the whole body; moreover, it can decrease the gluconeogenesis process in the liver [32]. Arginine has been used for regulation of blood pressure and the immune system [33]. Betaine has a role in the treatment of many human diseases including obesity, inflammation, diabetes, cancer, and Alzheimer’s [34].

In our study, we identified the presence of myo-inositol, sucrose, mannitol, fructose, and glucose in the polar plant extract. Glucose, fructose, sucrose, and mannitol have been reported in other species in the family Plantaginaceae [35]. Mannitol is a sugar alcohol that increases the tolerance of the plant to biotic and abiotic stress [36, 37]. We attribute the presence of this compound in *Anarrhinum pubescens* polar extract because the plant is growing in a stressful climatic condition.

The phenolic compound, gallic acid, and the alkaloids trigonelline and xanthine were identified in the aromatic region between δ5.0 and δ10.0. The presence of phenolic and alkaloid compounds in the family Plantaginaceae has been widely reported [23, 38]. Gallic acid was reported in other Plantaginaceae species like *Plantago coronopus* L. subsp. *coronopus* [39] and in *Plantago lanceolata* [40]. Gallic acid has been reported to have antioxidant and neuroprotective activities [41]. It was shown to improve gut microbe activity and positively modify the immune response [42], along with anti-cancer and anti-inflammatory properties [43]. Trigonelline is well known for its medicinal activity against diabetes and nervous system diseases [44, 45].

Vitamins and organic acids like choline and acetic acid have been identified in the plant extract. Choline is an essential nutrient for humans and its deficiency was shown to cause liver damage [46]. It is involved in lipoproteins, blood, and membrane lipid structure, and enhances liver function, neurodevelopment, and muscle movements [47].

### In vitro regeneration

Plant tissue culture techniques are ideal tools for conservation and propagation of rare and threatened plants. Many endangered plants have been conserved using different techniques like micropropagation and organogenesis through callus culture [48]. In the present work, the effect of different concentrations of BAP or Kn, in combination with NAA on micropropagation via nodal cuttings and leaf explants was investigated, and the data showed that BAP-treated cultures were significantly superior in de novo shoot induction and proliferation. BAP was the best cytokinin in shoot induction and proliferation from different plants and explants, e.g., *Bambusa balcooa* nodal cuttings [49], *Punica granatum* leaf explant [50], and in plant regeneration from *Bauhinia variegate* seeds [51]. In the family Plantaginaceae, BAP was more effective than Kn for shoot multiplication from *Bacopa monnieri* and *Plantago asiatica* in vitro cultures [52, 53]. A combination of BAP and NAA was more effective in organogenic shoots proliferation of *Mondia whitei* [54].

In this study, MS-B_5_ medium supplemented with IAA was the best in adventitious roots induction (85%) compared to MS-B_5_ hormone free medium. IAA has been found to play a central role in adventitious root formation in plants [55, 56]. Genes responsible for adventitious root formation in lotus seedling was regulated by IAA [57]. IAA was the most effective auxin in adventitious roots formation in apples [58].

### Genetic fidelity assessment of regenerated plants

Genetic variation is expected in in vitro regenerated plants, i.e., somaclonal variation [59]; so, it is important to assess the genetic stability of regenerated plants. In this work, RAPD and ISSR molecular markers were used to evaluate the genetic fidelity of the micropropagated clones. RAPD primers showed 0.97 to 0.98 similarity between the mother and the in vitro regenerated plants, while ISSR-based genomic analysis indicated a similarity index of 0.92–0.95 between the mother and the in vitro regenerated plants. Assessment of genetic stability using DNA molecular markers is a rapid and reliable method for determining genetic variation among and within plant species [60, 61]. RAPD and ISSR molecular markers were efficient in assessment of the genetic diversity in other members of the family Plantaginaceae, e.g., *Plantago ovata* [55] and *Plantago major* [62]. RAPD analysis has been used to evaluate genetic diversity within the wild *Anarrhinum pubescens* population [63].

## Conclusions

Metabolic profiling of the *A. pubescens* wild plant shoot has been performed in this study which can help in chemotaxonomy and in discovering new medicinal/nutritional uses of the plant shoots. We also developed an efficient, reproducible, and rapid protocol for the in vitro regeneration of the plant for the purpose of conserving its genetic resources as the resulting clones exhibited a high degree of genetic similarity to the mother plant.

## Data Availability

All data generated or analyzed during this study are included in this published article.

## Abbreviations

NMR: Nuclear Magnetic Resonance
HSQC: Heteronuclear Single Quantum Coherence
BAP: 6-Benzylaminopurine
NAA: Naphthaleneacetic acid
Kn: Kinetin
2,4-D: 2,4 dichlorophenoxy acetic acid
RAPD: Random Amplified Polymorphic DNA
ISSR: Inter Simple Sequence Repeat
MS-B5: Murashige and Skoog Medium with B5 vitamins

## References

[1] El-Husseini N, Abd El-Ghanii MM, El-Naggar SI (2008). Biogeography and diversity of the tubiflorae in Egypt. Pol Bot J.

[2] Boulos L (2009). Flora of Egypt. Checklist.

[3] Zahran MA, Wafaa AM, Samy AA, Omran GN (2015). Endemic species in Sinai Peninsula, Egypt, with particular reference to Saint Katherine protectorate: I- ecological features. J Environ Sci.

[4] Shaltout KH, Al-Sodany YM, Eid EM, Heneidy SZ, Taher MA (2020). Vegetation diversity along the altitudinal and environmental gradients in the main wadi beds in the mountainous region of South Sinai, Egypt. Journal of Mountain Science.

[5] Omar K (2017). *Anarrhinum pubescens*. The IUCN red list of threatened species 2017.

[6] Moustafa AA, Zaghloul MS (1996). Environmental and vegetation in the mountane Saint Catherine area, South Sinai. Egypt. J Arid Environ.

[7] Walter KS, Gillett HJ (1998). 1997 IUCN Red list of threatened plants.

[8] Mahran E, Hosny M, El-Hela A, Boroujerdi A (2019). New iridoid glycosides from *Anarrhinum pubescens*. Nat Prod Res.

[9] Mahran E, Morlock GE, Keusgen M (2020). Guided isolation of new iridoid glucosides from *Anarrhinum pubescens* by high-performance thin-layer chromatography-acetylcholinesterase assay. J Chromatogr A.

[10] Tundis R, Loizzo MR, Menichini F, Statti GA, Menichini F (2008). Biological and pharmacological activities of iridoids: recent developments. Mini Rev Med Chem.

[11] Joubouhi C, Tamokou J, Ngnokam D, Voutquenne-Nazabadioko L, Kuiate J (2017). Iridoids from Canthium subcordatum iso-butanol fraction with potent biological activities. BMC Complement Altern Med.

[12] Dawidar AM, Esmirly ST, ASM AL-H, Jakupovic J, Abdel-Mogib M (1989). Two iridoid glucoside esters from *Anarrhinum orientale*. Phytochemistry.

[13] Salah El Dine R, Abdel Monem A, El-Halawany A, Hattori M, Abdel-Sattar E (2011). HCV-NS3/4A protease inhibitory iridoid glucosides and dimeric foliamenthoic acid derivatives from *Anarrhinum orientale*. J Nat Prod.

[14] Shree M, Lingwan M, Masakapalli SK (2019). Metabolite profiling and metabolomics of plant systems using 1H NMR and GC-MS. OMICS-Based Approaches Plant Biotechnol.

[15] Vinoth A, Ravindhran R (2013). *In vitro* propagation- a potential method for plant conservation. Int J comput Algorithm.

[16] Shahzad A, Saeed T, Shahid M, Shahzad A, Malik A, Sahai A (2013). *In vitro* conservation protocols for some rare medicinal plant species. Recent trends in biotechnology and therapeutic applications of medicinal plants.

[17] Engelmann F (2011). Use of biotechnologies for the conservation of plant biodiversity. In Vitro Cell Dev Biol Plant.

[18] Makowczynska J, Andrzejewska-Golec E (2009). Micropropagation of Plantago maritima L.-a vanishing species in Poland. Acta Societatis Botanicorum Poloniae.

[19] Haque SM, Chakraborty A, Dey D, Mukherjee S, Nayak S, Ghosh B (2017). Improved micropropagation of *Bacopa monnieri* (L.) Wettst. (Plantaginaceae) and antimicrobial activity of *in vitro* and *ex vitro* raised plants against multidrug-resistant clinical isolates of urinary tract infecting (UTI) and respiratory tract infecting (RTI) bacteria. Clin Phytosci.

[20] Sarsan VA, Cripps R, Ramsay MM, Atherton C, McMichen M, Prendergast G, Rowntree JK (2006). Conservation in vitro of threatened plants- progress in the past decade. In Vitro Cell Dev Biol Plant.

[21] Joshi P, Dhawan V (2007). Assessment of genetic fidelity of micropropagated *Swertiachirayita* plantlets by ISSR Marker Assay. Biol Plant.

[22] Kumar A, Prakash K, Sinha RK, Kumar N (2013). In vitro plant propagation of *Catharanthus roseus* and assessment of genetic fidelity of micropropagated plants by RAPD marker assay. Appl Biochem Biotechnol.

[23] Tlili H, Hanen N, Ben Arfa A, Neffati M, Boubakri A, Buonocore D, Dossena M, Verri M, Doria E (2019). Biochemical profile and *in vitro* biological activities of extracts from seven folk medicinal plants growing wild in southern Tunisia. PloS one..

[24] Saracoglu I, Suleimanov T, Pashaeva N, Dogan Z, Inoue M, Nakashima K (2020). Iridoids from Veronica crista-galli from the Flora of Azerbaijan. Chem Nat Compd.

[25] Kim HK, Choi YH, Verpoorte R (2011). NMR-based plant metabolomics: where do we stand, where do we go?. Trends Biotechnol.

[26] Murashige T, Skoog F (1962). A revised medium for rapid growth and bioassay with tobacco tissue culture. Plant Physiol.

[27] Gamborg OL, Miller RA, Ojima K (1968). Nutrient requirements of suspension culture of soybean root cells. Exp Cell Res.

[28] Jaccard P (1908). Nouvelles recherches sur la distribution florale. Bull Soc Vaudoise Sci Nat.

[29] Henderson PA, Seaby RMH (1999). CAP (community analysis package).

[30] Patel MK, Mishra A, Jha B (2016). Non-targeted metabolite profiling and scavenging activity unveil the nutraceutical potential of psyllium (Plantago ovata Forsk). Front Plant Sci.

[31] Osmachko АP, Kovaleva AM, Goryacha ОV, Avidzba YN (2016). Amino acid composition of *Veronica teucrium* L. herb. Der Pharma Chemica.

[32] Yoshizawa F (2012). New therapeutic strategy for amino acid medicine: notable functions of branched chain amino acids as biological regulators. J Pharmacol Sci.

[33] McRae MP (2016). Therapeutic benefits of l-arginine: an umbrella review of meta-analyses. J Ciropr Med.

[34] Zhao G, He F, Wu C, Li P, Li N, Deng J, Zhu G, Ren W, Peng Y (2018). Betaine in inflammation: mechanistic aspects and applications. Front Immunol.

[35] Lohaus G, Schwerdtfeger M (2014). Comparison of sugars, iridoid glycosides and amino acids in nectar and phloem sap of *Maurandya barclayana*, *Lophospermum erubescens*, and *Brassica napus*. PLoS One.

[36] Stoop JM, Williamson JD, Pharr DM (1996). Mannitol metabolism in plants: a method for coping with stress. Trend Plant Sci.

[37] Patel TK, Williamson JD (2016). Mannitol in plants, fungi, and plant–fungal interactions. Trend Plant Sci.

[38] Adom MB, Taher M, Mutalabisin MF, Amri MS, Kudos MB, Sulaiman MW, Sengupta P, Susanti D (2017). Chemical constituents and medical benefits of Plantago major. Biomed Pharmacother.

[39] Teğin I, Canpolat G, Fidan M (2018) The antioxidant capacity, total phenolic content and phenolic compounds of *Plantago coronopus* L. subsp. *coronopus* in naturally distributed in Akdoğmuş-Siirt. In: 2018 2nd International Symposium on Multidisciplinary Studies and Innovative Technologies (ISMSIT). IEEE, Ankara, pp 1–4

[40] Beara IN, Lesjak MM, Orčić DZ, Simin NĐ, Četojević-Simin DD, Božin BN, Mimica-Dukić NM (2012). Comparative analysis of phenolic profile, antioxidant, anti-inflammatory and cytotoxic activity of two closely-related Plantain species: *Plantago altissima* L. and Plantago lanceolata L. LWT-Food Sci Technol.

[41] Daglia M, Di Lorenzo A, Nabavi SF, Talas ZS, Nabavi SM (2014). Polyphenols: well beyond the antioxidant capacity: gallic acid and related compounds as neuroprotective agents: you are what you eat!. Curr Pharm Biotechnol.

[42] Zhou D, Yang Q, Tian T, Chang Y, Li Y, Duan LR, Li H, Wang SW (2020). Gastroprotective effect of gallic acid against ethanol-induced gastric ulcer in rats: involvement of the Nrf2/HO-1 signaling and anti-apoptosis role. Biomed Pharmacothe.

[43] Kang DY, Sp N, Jo ES, Rugamba A, Hong DY, Lee HG, Yoo JS, Liu Q, Jang KJ, Yang YM (2020). The inhibitory mechanisms of tumor PD-L1 expression by natural bioactive gallic acid in non-small-cell lung cancer (NCLC) cells. Cancers.

[44] Zhou J, Chan L, Zhou S (2012). Trigonelline: a plant alkaloid with therapeutic potential for diabetes and central nervous system disease. Curr med chem.

[45] Folwarczna J, Janas A, Pytlik M, Cegieła U, Śliwiński L, Krivošíková Z, Štefíková K, Gajdoš M (2016). Effects of trigonelline, an alkaloid present in coffee, on diabetes-induced disorders in the rat skeletal system. Nutrients..

[46] Mehedint MG, Zeisel SH (2013). Choline’s role in maintaining liver function: new evidence for epigenetic mechanisms. Curr Opin Clin Nutr Metab Care.

[47] Ueland PM (2011). Choline and betaine in health and disease. J Inherit Metab Dis.

[48] Fay MF (1992). Conservation of rare and endangered plants using *in vitro* methods. In Vitro Cell Dev Biol Plant.

[49] Rajput BS, Jani M, Ramesh K, Manokari M, Jogam P, Allini VR, Kher MM, Shekhawat MS (2020). Large-scale clonal propagation of *Bambusa balcooa* Roxb.: an industrially important bamboo species. Ind Crop Prod.

[50] Verma V, Zinta G, Kanwar K (2021). Optimization of efficient direct organogenesis protocol for Punica granatum L. cv. Kandhari Kabuli from mature leaf explants. In Vitro Cell Dev Biol Plant.

[51] Singh BMS (2020) Effects of cytokinin on *in vitro* propagation of *Bauhinia variegata* L. Eur J Biol Biotechnol 1(6). 10.24018/ejbio.2020.1.6.123

[52] Haque SM, Chakraborty A, Dey D, Mukherjee S, Nayak S, Ghosh B (2017). Improved micropropagation of *Bacopa monnieri* (L.) Wettst (Plantaginaceae) and antimicrobial activity of *in vitro* and *ex vitro* raised plants against multidrug-resistant clinical isolates of urinary tract infecting (UTI) and respiratory tract infecting (RTI) bacteria. Clin Phytosci.

[53] Makowczynska J, Andrzejewska-Golec E (2003) Micropropagation of *Plantago asiatica* L. through culture of shoot-tips. Acta Soc Bot Pol 72:191-194.

[54] Patricia D, Stephen B, John A (2021) Shoot organogenesis from leaf discs of the African ginger (*Mondia whitei* (Hook. f.) Skeels), an endangered medicinal plant. In Vitro Cell Dev Biol Plant 57:1–6

[55] Negi S, Sukumar P, Liu X, Cohen JD, Muday GK (2010). Genetic dissection of the role of ethylene in regulating auxin-dependent lateral and adventitious root formation in tomato. Plant J.

[56] Zhubing H, Shuyan L (2020). Comparative transcriptome analysis revealed the cooperative regulation of sucrose and IAA on adventitious root formation in lotus (Nelumbo nucifera Gaertn). BMC Genomics.

[57] Libao C, Runzhi J, Jianjun Y, Xiaoyong X, Haitao Z, Shuyan L (2018). Transcriptome profiling reveals an IAA-regulated response to adventitious root formation in lotus seedling. Z Naturforsch C.

[58] De Klerk GJ, Ter Brugge J, Marinova S (1997). Effectiveness of indoleacetic acid, indolebutyric acid and naphthaleneacetic acid during adventitious root formation *in vitro* in Malus ‘Jork 9’. Plant Cell Tissue Organ Cult.

[59] Krishna H, Alizadeh M, Singh D, Singh U, Chauhan N, Eftekhari M, Sadh RK (2016). Somaclonal variations and their applications in horticultural crops improvement. 3 Biotech.

[60] Kumar P, Gupta VK, Misra AK, Modi DR, Pandey BK (2009). Potential of molecular markers in plant biotechnology. Plant Omics.

[61] Nadeem MA, Nawaz MA, Shahid MQ, Doğan Y, Comertpay G, Yıldız M, Hatipoğlu R, Ahmad F, Alsaleh A, Labhane N, Özkan H (2018). DNA molecular markers in plant breeding: current status and recent advancements in genomic selection and genome editing. Biotechnol Biotechnol Equip.

[62] Keivani M, Mehregan I, Albach DC (2020). Genetic diversity and population structure of *Plantago major* (Plantaginaceae) in Iran. Iran J Bot.

[63] Ahmed AM, Ghaly ON (2014). Fingerprint’s documentation of endemic plant species to Saint Katherine protectorate south Sinai-Egypt. Environ. Sci.

